# An fMRI Study of Intra-Individual Functional Topography in the Human Cerebellum

**DOI:** 10.3233/BEN-2010-0268

**Published:** 2010-08-16

**Authors:** Catherine J. Stoodley, Eve M. Valera, Jeremy D. Schmahmann

**Affiliations:** ^1^Cognitive/Behavioral Neurology UnitMassachusetts General Hospital and Harvard Medical SchoolBostonMAUSA; ^2^Laboratory for Neuroanatomy and Cerebellar NeurobiologyMassachusetts General Hospital and Harvard Medical SchoolBostonMAUSA; ^3^Ataxia UnitDepartment of NeurologyMassachusetts General Hospital and Harvard Medical SchoolBostonMAUSA; ^4^Athinoula A. Martinos Center for Biomedical Imaging and Department of PsychiatryMassachusetts General Hospital and Harvard Medical SchoolBostonMAUSA

**Keywords:** Cerebellum, topography, functional MRI, cognition, motor

## Abstract

Neuroimaging studies report cerebellar activation during both motor and non-motor paradigms, and suggest a functional topography within the cerebellum. Sensorimotor tasks activate the anterior lobe, parts of lobule VI, and lobule VIII, whereas higher-level tasks activate lobules VI and VII in the posterior lobe. To determine whether these activation patterns are evident at a single-subject level, we conducted functional magnetic resonance imaging (fMRI) during five tasks investigating sensorimotor (finger tapping), language (verb generation), spatial (mental rotation), working memory (N-back), and emotional processing (viewing images from the International Affective Picture System). Finger tapping activated the ipsilateral anterior lobe (lobules IV-V) as well as lobules VI and VIII. Activation during verb generation was found in right lobules VII and VIIIA. Mental rotation activated left-lateralized clusters in lobules VII-VIIIA, VI-Crus I, and midline VIIAt. The N-back task showed bilateral activation in right lobules VI-Crus I and left lobules VIIB-VIIIA. Cerebellar activation was evident bilaterally in lobule VI while viewing arousing vs. neutral images. This fMRI study provides the first proof of principle demonstration that there is topographic organization of motor execution vs. cognitive/emotional domains within the cerebellum of a single individual, likely reflecting the anatomical specificity of cerebro-cerebellar circuits underlying different task domains. Inter-subject variability of motor and non-motor topography remains to be determined.

## 1. Introduction

The cerebellum has long been recognized as essential for the control of posture and movement [20,35]. It is now apparent that lesions of the cerebellum may produce impairments in working memory, attention, linguistic and spatial processing, and emotional modulation, first described as the cerebellar cognitive affective syndrome (CCAS) [50,81]. The CCAS is postulated to result from disruptions in the distributed neural circuits linking the cerebellum with associative and limbic/paralimbic regions of the cerebral hemispheres, producing dysmetria of thought analogous to dysmetria of motor control [71,73,74,81].

Anatomical [43,54,77,79,80], physiological [3,85–87], behavioral [5,67], and clinical studies [22,75,81, 82] indicate that there is topographic arrangement of function in the cerebellum [for review, see [89]]. Sensorimotor domains are represented in the anterior lobe (mostly lobules IV and V), with face representation

ISSN 0953-4180/10/$27.50 © 2010 – IOS Press and the authors. All rights reserved extending into medial lobule VI, as well as in lobule VIII, which is regarded as the second somatosensory representation [97]. In contrast, the hemispheric component of lobule VII (Crus I, Crus II and lobule VIIB) does not receive motor inputs, and was silent in the sensorimotor physiological experiments of Snider and colleagues [86,87]. The cerebellar hemispheres, largely comprised of lobules VI and VII, have expanded massively through evolution [4,52,96], along with the cerebral association areas and neodentate nucleus [49, 96], and are linked with non-motor areas of the cerebral cortex [79].

The results of functional neuroimaging studies in healthy subjects also provide support for cerebellar functional topography, as summarized in our recent meta-analysis [88]. Upper extremity sensorimotor tasks localize to the anterior lobe (lobule V), with a second focus in lobules VIIIA/B [14,28,29,68]. The right posterolateral cerebellum is active during language tasks, including verbal fluency, verb-for-noun generation, semantic judgment, and word stem completion [13,19,23, 26,30,33,38,51,53,55,56,59,65,66,70,83,91,98]. Spatial tasks, exemplified by line bisection, mental rotation and spatial transformation tasks, activate regions in lobules VI and Crus I (e.g. [8,25,42,64,94,95,99]). Working memory paradigms, such as the N-back task, activate bilateral regions in the posterior lobe [15,16,36, 45,92,93]. Cerebellar lobules VI and VII are active during executive function tasks, such as the Tower of London, random number generation and complex decision-making tasks [7,17,32,40,69]. Affective/emotional processing involves lobules VI and VII [6,27,34,46,48,61–63,90]. Furthermore, these findings are supported by recent studies indicating that the cerebellum can be divided into different regions based on its functional connectivity with sensorimotor vs. prefrontal and parietal cortices [2,31,44,58].

The results of the meta-analysis thus indicate that the cerebellar anterior lobe, extending into lobule VI, and lobule VIII are engaged during tasks involving overt motor processing (finger tapping, articulation), whereas cerebellar posterior lobe regions (particularly vermal and hemispheric components of lobules VI and VII) are active during language, spatial, executive function and affective processing [88]. However, meta-analyses are limited by the combination of data from many different studies, acquired on scanners of different strengths, while subjects completed different task paradigms, and that were analyzed with a variety of techniques and statistical thresholds. Because no study to date has investigated the activation patterns for these various tasks within individual subjects, the precise topographic ordering and possible overlap of these functional domains within the human cerebellum has not yet been determined. These data would provide important information necessary to understand the effects of focal cerebellar injury on sensorimotor function, cognition and emotion in individual patients. In the present study, therefore, we tested whether it is possible to establish intra-individual cerebellar topography by performing functional magnetic resonance imaging (fMRI) in a single subject during five different tasks designed to tap sensorimotor, language, spatial, working memory, and affective processing.

## 2. Methods

### 2.1. Subject

One healthy, right-handed adult male (aged 32 years, 2 months) with no history of neurological illness or injury participated in the study. The project was approved by the Institutional Review Board of the Massachusetts General Hospital and the subject provided written, informed consent. Handedness was confirmed by a score of 69 on the Edinburgh Handedness Inventory ($> 40$ indicates right-handedness; [57]).

### 2.2. Cognitive ability

To confirm that the subject's cognitive ability was in the normal range, the abbreviated form of the Wechsler Adult Intelligence Scale-III (WAIS-III; The Psychological Corporation, 1997) was administered. This standardized scale of intelligence includes eight subtests (Similarities, Arithmetic, Digit span, Information, Picture completion, Digit symbol and Block design) designed to measure both verbal and non-verbal cognitive ability.

### 2.3. Tasks

The subject was familiarized with the tasks before the fMRI session. The tapping task was run using Matlab 7.0.4 (Mathworks, Sherborn, MA) on a Sony Vaio laptop; all other tasks were presented using MacStim software (White Ant Occasional Publishing, West Melbourne, Australia) and run on a Mac iBook laptop. In the scanner, the subject completed five different tasks that were chosen because they reliably activate the cerebellum in imaging studies. These included:

#### 2.3.1. *Finger-tapping* (*sensorimotor processing*)

The subject was required to tap a button with his right index finger in time with cued beeps at a pace of 2 Hz. After an initial fixation period, instructions to "Tap along" were given for 3.5 s, followed by 15 s of cued tapping. "Stop" instructions were then followed by "Listen only" (2.5 s) instructions, which were followed by 15 s of the cuing beeps during which the subject rested. A 15 s fixation period separated the blocks. The subject repeated each block (tapping followed by listen only followed by fixation) 6 times, leading to a total run time of 5:36. Accuracy and response time data were measured.

#### 2.3.2. *N-back task* (*working memory, executive function*)

The N-back task required the subject to respond with a button press when the current letter on the screen was the same as that which appeared "N" items previously; an X-back task, in which the subject responded to the letter X, was used for comparison. In this study, we used a 2-back task. Following initial fixation of 20 s, "Respond to X" appeared for 4 s, followed by 16 letters (0.2 s presentation with 1.8 s inter-stimulus-interval [ISI]). The subject responded by pressing a button with his right index finger when the letter "X" appeared, which was approximately 25% of the time. Following a 20 s fixation period, "2 back" appeared for 4 s, to cue the subject to perform the 2-back task. Again, 16 letters appeared in succession and the subject was asked to press a button when the letter presented was the same as that appearing 2-back (approximately 25% of trials). The fixation-X back-fixation-2 back sequence repeated three times, leading to a total run time of 5:36. Two runs of the task were performed. Performance accuracy and response times were recorded.

#### 2.3.3. *Verb generation* (*language*)

In this task, the subject covertly generated a verb when presented with a concrete noun (e.g., drink ♢ beer). After an initial instruction period, "Read only" appeared for 5 s, followed by 10 nouns, each of which appeared for 2 s with no ISI. During this time, the subject was instructed to read the nouns as they appeared. After a 5 s fixation period, "Generate a verb" appeared for 5 s, cuing the verb generation task. Again, 10 nouns appeared and the subject covertly generated a verb for each noun. Each run comprised initial instructions and four blocks of read only interleaved with four blocks of verb generation, leading to a total run time of 4:18. The same stimuli were used for the read only and verb generation conditions, appearing in randomized order. The subject performed two runs of the task, and after the scan session provided verbs for a sample set of the nouns presented in the study as a measure of accuracy.

#### 2.3.4. *Mental rotation* (*visual-spatial processing*)

The subject was required to judge whether a rotated letter was presented in its normal form or as a mirror image by pressing the button under his right index finger if the letter was in its normal orientation, and the button under his right middle finger if the letter was mirror-oriented. After 18 s of initial instructions, blocks of non-rotated and rotated letters (F, G, J and R) alternated. The rotated letters appeared at angles of 40, 120, 200, and 280 degrees. Each block comprised 8 black letter stimuli which appeared on a gray background for 1500 ms with an ISI of 1500 ms. There were 6 s rest periods between blocks. Each run comprised four blocks with non-rotated letters alternating with four blocks with rotated letters, leading to a total run time of 4:18. The subject completed two runs of the task. Behavioral performance in the scanner was analyzed for accuracy and response times.

#### 2.3.5. *Viewing of pictures from the International Affective Picture System* (*IAPS [47]; affective processing*)

These images are similar with regard to visual features (complexity, color, etc.) but differ in emotional content. The subject viewed images rated as "highly arousing" (average standard valency for negative images was 2.9, and for positive images 6.7; arousal ratings were 6.4 and 6.3, respectively) and "neutral" (average valency, 5.2; average arousal rating, 2.6) while in the scanner. Images with extreme arousal content were excluded. The subject completed one run of the task. After an initial instruction period of 11s, 8 blocks comprised of 8 neutral images alternated with 8 blocks of 8 arousing images, with 20 s of fixation between the blocks. The images appeared for 2 s with an ISI of 500 ms, leading to a total run time of 5:31. After the scan session, the subject viewed a subset of the images and rated them on a scale of 1-9 to assess the degree to which he found them negative/positive or neutral/arousing.

### 2.4. *fMRI scanning*

Scanning was performed at the Athinoula A. Martinos Center for Biomedical Imaging using a 3T TimTrio (Siemens, Erlanger, Germany) MRI with a 12-channel head coil. During scanning, the subject's head was immobilized using tight but comfortable foam padding. Stimuli were presented via a liquid crystal display (LCD) projector onto a screen located in the scanner bore and viewed through a mirror attached to the head coil; auditory stimuli were presented through headphones. The subject underwent a sagittal T1-weighted structural MR scan (magnetization prepared rapid gradient echo, MPRAGE), with 128 1.33 mm-thick slices, $1.3 \times 1.0 \times 1.3$ mm voxel size, TR 2530 ms, TE 3.39 ms, flip angle $7^{\circ}$, and field of view (FOV) $256 \times 256$ mm. Functional echo-planar imaging (EPI) runs were performed with the following parameters: 42 interleaved axial slices at a TR 2900 ms, TE 30 ms, voxel size $3 \times 3 \times 3$ mm, flip angle $90^{\circ}$, FOV 288 mm. One run each of the tapping (112 measurements) and affective (110 measurements) tasks were run. The subject completed two runs of the verb generation (85 measurements per run), mental rotation (85 measurements per run) and 2N-back task (112 measurements per run).

### 2.5. *Data analysis*

Analysis of fMRI data was performed using Statistical Parametric Mapping, version 5 (SPM5; Wellcome Department of Cognitive Neurology, London). Data were realigned for motion correction by registration to the mean image, normalized to the SPM5 EPI template using affine transformation, and smoothed using an 8 mm full width half maximum filter. Each block of trials was modeled using a boxcar function convolved with a canonical hemodynamic response function. General linear modeling (GLM) was employed to form statistical parametric maps of the T-statistic. The primary contrasts were as follows: 1. Tapping vs. Listen only; 2. 2-back vs. X-task; 3. Verb generation vs. Read only; 4. Rotated vs. Non-rotated letters; 5. Arousing vs. Neutral images.

The resulting T-maps showed a large range of maximum T values between the tasks (likely due to the degree to which the task of interest and the comparison condition were related). The maximum T values ranged from a low of 6.98 for the mental rotation task to a high of 20.58 for the tapping task. Because our goal was to compare the spatial localization of the cerebellar activation patterns, an uncorrected threshold of $p < 0.005$ was used for the task with the weakest level of cerebellar activation (viewing IAPS images; this threshold corresponded to a T-value of 2.71). To provide relative comparisons between the tasks, the other maps were thresholded using a proportional T-value scale. We based our relative thresholding on the ratio between the maximum overall T of 7.63 for the IAPS task and the minimum cerebellar T-value of 2.71 in the IAPS task, which gave a ratio of 2.8. Therefore, each maximum T-value was divided by 2.8 to obtain the threshold T-value for each task map. Thus, the following thresholds were used for the other four tasks: tapping, $T > 7.31$, which corresponded to family-wise error (FWE)-corrected $p < 0.0001$; verb generation, $T > 3.99$, which corresponded to a false discovery rate (FDR)-corrected $p < 0.001$; N-back, $T > 3.65$, which corresponded to FDR-corrected $p < 0.002$; and mental rotation, $T > 2.48$, which corresponded to an uncorrected $p = 0.007$. For all analyses, clusters meeting an extent threshold of $k > 5$ and $p < 0.05$ (corrected) are reported unless otherwise noted.

The MRI Atlas of the Human Cerebellum [76] and the subject's T1 MPRAGE scan were used to localize activation to lobules in the cerebellum. Activation patterns in the cerebral hemispheres were localized using the Anatomy Toolbox for SPM [21]. The subject's T1 MPRAGE scan was normalized to MNI space in SPM5 and used as the underlay for the SPM{T} maps. The activation patterns for all tasks were visualized with MRIcron software (http://www.sph.sc.edu/comd/rorden/mricron/).

## 3. Results

### 3.1. *Cognitive ability*

Performance on the WAIS-III indicated that the subject performed in the normal to superior range on all subtests administered (subtest scores have a mean of 10 and standard deviation of 3; the subject scored a low of 8 on one subtest [picture completion], with all other subtest scores ranging from 14–17). The subject's estimated full-scale IQ was 134, with a verbal IQ of 134 and performance IQ of 125.

### 3.2. *Behavioral performance*

#### 3.2.1. *Tapping*

Apart from two prolonged tap responses during the first tapping block, the subject correctly tapped along to the 2 Hz stimuli, and no erroneous taps were made during the "Listen only" condition. Median tap times for each of the six tapping blocks were 502.6 ms, 497.14 ms, 502.92 ms, 495.96 ms, 503.50 ms and 501.45 ms (tapping should take place every 500 ms according to the pacing cue). The overall median RT was 501.07 ms, indicating that the subject very accurately performed the task.

Table 1
Tapping vs. Listen only

| Cluster | Cluster size | Location | T-value | MNI coordinates | | |
|---|---|---|---|---|---|---|
| 1 | 954 | L Pericentral cortex | 20.58 | −42 | −15 | 48 |
| | | L Pericentral cortex | 17.42 | −45 | −18 | 60 |
| | | L Pericentral cortex | 17.37 | −33 | −24 | 48 |
| 2 | 327 | **R Cerebellum V/VI** | 17.38 | 24 | −51 | −24 |
| | | **R Cerebellum IV** | 9.66 | 3 | −51 | −6 |
| | | **R Cerebellum VI** | 9.64 | 3 | −66 | −21 |
| 3 | 151 | **R Cerebellum VIIIA** | 12.21 | 15 | −66 | −48 |
| | | **R Cerebellum VIIIB** | 9.04 | 27 | −54 | −57 |
| | | **R Cerebellum VIIIA** | 7.36 | 36 | −48 | −54 |
| 4 | 24 | L Middle cingulate gyrus | 9.06 | −9 | −27 | 45 |
| 5 | 54 | L Rolandic operculum / insula | 8.48 | −45 | −15 | 12 |
| | | L Superior temporal gyrus | 8.46 | −54 | −24 | 9 |
| 6 | 16 | Medial frontal gyrus | 7.88 | −3 | −6 | 54 |
| 7 | 6 | Precuneus | 7.19 | 0 | −72 | 60 |

Key: $R$ = Right; $L$ = Left. MNI coordinates = x, y, z coordinates of cluster peaks. Coordinates meeting FWE-corrected threshold of $p < 0.0001$ and cluster-level threshold of $p < 0.05$ (corrected) are reported.

*3.2.2. N-back*

On the X-task, the subject performed at 100% accuracy with a median response time (RT) of 455 ms; on the 2-back task, accuracy was 84% with a median RT of 442 ms.

*3.2.3. Verb generation*

In the post-scan test, the subject scored 39/40 correct (97.5%) on the verb generation task, indicating that he generated suitable responses during the task.

*3.2.4. Mental rotation*

Mean overall accuracy on the task was 93.5%, with a median RT 781 ms. Accuracy and median RT for the upright letters was 95.1% and 764 ms, and for rotated letters accuracy was 91.9% with a median RT of 752 ms.

*3.2.5. IAPS pictures*

The subject's post-scan ratings of 16 selected IAPS images did not differ from the norm scores for those images with regard to both valency and arousal. The subject's average z-scores were $-0.02$ for valency and $-0.42$ for arousal, based on the means and standard deviations provided by the IAPS scale for the images tested. This indicates that the images triggered normal emotional responses in our subject.

*3.3. Functional imaging*

Tables 1–5 show the whole-brain activation patterns for the five different tasks, with cerebellar activations highlighted in bold. The cortical activation patterns were consistent with those found in previous functional imaging studies utilizing these measures. Figure 1 shows the cerebellar activation patterns for the different tasks in coronal, axial and sagittal slices. Activation patterns are overlaid onto the subject's normalized T1-weighted MR scan.

*3.3.1. Tapping*

The contrast of Finger tapping vs. Listen only showed activation focused in the ipsilateral cerebellum (Table 1). Two large, right-lateralized cerebellar clusters were evident: one in the anterior lobe in lobules IV and V, and extending into lobule VI, and a second located in lobules VIIIA and VIIIB. This was the only cerebellar activation that was evident at the midline ($x = 0$, see Fig. 1C); the majority of non-motor activation patterns only emerged more laterally in the hemispheres (e.g., around $\times$ 24).

In the cerebral cortex there was a large activation cluster in left sensorimotor cortices (including Brodmann Areas [BAs] 3, 4 and 6). Other left-lateralized cortical clusters were found in the cingulate gyrus, the operculum/insula/superior temporal gyrus, and the medial frontal gyrus.

*3.3.2. N-back*

The contrast of the 2-back vs. X-task resulted in cerebellar activation bilaterally, involving a right-sided cluster in cerebellar lobules VI through Crus I, and a left-sided cluster with peaks in lobules VIIB and VIIIA (Table 2). In the left cerebellar hemisphere, the activation was more anterior, whereas in the right cerebellar hemisphere, the activation extended along lobule VI and Crus I in the anterior to posterior direction (see Fig. 1C).

Table 2
N-back vs. X-task

| Cluster | Cluster size | Location | T-value | MNI coordinates | | |
|---|---|---|---|---|---|---|
| 1 | 1124 | L Middle frontal gyrus | 10.27 | −54 | 21 | 36 |
| | | L Superior frontal gyrus | 9.16 | −27 | 6 | 69 |
| | | L Precentral gyrus | 6.99 | −54 | 6 | 45 |
| 2 | 672 | R Middle frontal gyrus | 8.97 | 39 | 6 | 66 |
| | | R Middle frontal gyrus | 8.50 | 45 | 42 | 36 |
| | | R Middle frontal gyrus | 7.59 | 51 | 15 | 51 |
| 3 | 134 | **R Cerebellum, Crus I** | 7.53 | 42 | −51 | −33 |
| | | **R Cerebellum, VI** | 5.55 | 33 | −63 | −30 |
| 4 | 478 | Precuneus | 7.31 | 15 | −69 | 48 |
| | | R Postcentral gyrus | 6.52 | 45 | −42 | 66 |
| | | Precuneus | 6.14 | −9 | −69 | 41 |
| 5 | 202 | Medial frontal gyrus | 7.10 | −6 | 9 | 54 |
| | | Superior frontal gyrus | 6.13 | 6 | 18 | 51 |
| | | Middle cingulate gyrus | 5.92 | −9 | 21 | 36 |
| 6 | 184 | Insula | 6.89 | 30 | 24 | 9 |
| | | Basal ganglia, Putamen | 3.99 | 27 | 3 | 18 |
| 7 | 27 | L Middle temporal gyrus | 6.61 | −69 | −6 | −9 |
| 8 | 99 | **L Cerebellum, VIIB** | 6.51 | −45 | −42 | −42 |
| | | **L Cerebellum, VIIIA** | 5.83 | −33 | −39 | −45 |
| 9 | 561 | Brainstem, Red nucleus | 6.12 | −3 | −27 | −12 |
| | | Brainstem, Red nucleus | 5.95 | 6 | −27 | −15 |
| | | Thalamus, pulvinar | 5.86 | −9 | −24 | 12 |
| 10 | 39 | R Middle frontal gyrus | 5.51 | 33 | 42 | 15 |
| 11 | 70 | L Inferior parietal lobule | 5.47 | −33 | −48 | 48 |
| | | L Inferior parietal lobule | 5.24 | −39 | −39 | 39 |

Key: $R$ = Right; $L$ = Left. MNI coordinates = x, y, z coordinates of cluster peaks. Coordinates meeting FDR-corrected $p < 0.002$ and cluster-level $p < 0.05$ (corrected) are reported.

Table 3
Verb Generation vs. Reading Nouns

| Cluster | Cluster size | Location | T-value | MNI coordinates | | |
|---|---|---|---|---|---|---|
| 1 | 3018 | L Precentral gyrus | 11.23 | −48 | 0 | 33 |
| | | L Precentral gyrus | 9.83 | −54 | 9 | 39 |
| | | L Supplementary motor area | 8.74 | −6 | 12 | 57 |
| 2 | 1109 | L Supramarginal gyrus | 10.34 | −51 | −45 | 24 |
| | | L Superior parietal lobule | 8.36 | −33 | −66 | 51 |
| | | L Inferior parietal lobule | 7.60 | −45 | −54 | 51 |
| 3 | 50 | R Precentral gyrus | 7.69 | 60 | 3 | 45 |
| 4 | 96 | R Supramarginal gyrus | 6.41 | 69 | −24 | 18 |
| | | R Superior temporal gyrus | 6.28 | 69 | −39 | 12 |
| | | R Middle temporal gyrus | 4.37 | 60 | −24 | −3 |
| 5 | 368 | **R Cerebellum VIIB** | 6.15 | 39 | −63 | −54 |
| | | **R Cerebellum VI / Crus I** | 6.02 | 36 | −51 | −33 |
| | | **R Cerebellum VIIIA** | 5.94 | 33 | −69 | −54 |
| 6 | 32 | Thalamus | 5.74 | 9 | −6 | 9 |
| | | Parahippocampal gyrus | 5.24 | 15 | −9 | −18 |
| 7 | 45 | Caudate, body | 5.50 | 18 | 21 | 0 |
| | | Caudate, body | 4.80 | 18 | 18 | 9 |
| | | R Anterior cingulate gyrus | 4.47 | 15 | 30 | 27 |
| 8 | 35 | Thalamus | 5.01 | 15 | −21 | 15 |
| | | Thalamus | 4.07 | 18 | −30 | 9 |
| 9 | 19 | **R Cerebellum, anterolateral white matter** | 4.64 | 24 | −33 | −39 |
| 10 | 21 | Thalamus | 4.45 | −6 | −21 | 15 |
| | | Thalamus | 4.11 | −15 | −18 | 15 |

Key: $R$ = Right; $L$ = Left. MNI coordinates = x, y, z coordinates of cluster peaks. Coordinates meeting FDR-corrected threshold of $p < 0.001$ and cluster-level threshold of $p <$ 0.05 (corrected) are reported.

Table 4
Mental rotation vs. upright letters

| Cluster | Cluster size | Location | T-value | MNI coordinates | | |
|---|---|---|---|---|---|---|
| 1 | 1730 | R Postcentral gyrus | 6.98 | 39 | −45 | 66 |
| | | R Inferior temporal gyrus | 6.11 | 45 | −72 | −9 |
| | | R Superior occipital gyrus | 5.30 | 27 | −96 | 12 |
| 2 | 814 | R Superior frontal gyrus | 6.05 | 33 | 3 | 66 |
| | | R Middle frontal gyrus | 5.67 | 45 | 6 | 57 |
| | | R Middle frontal gyrus | 5.36 | 54 | 36 | 30 |
| 3 | 375 | L Middle frontal gyrus | 5.54 | −45 | 54 | 9 |
| | | L Middle frontal gyrus | 5.33 | −48 | 45 | 21 |
| | | L Middle frontal gyrus | 4.71 | −48 | 33 | 36 |
| 4 | 989 | L Inferior occipital gyrus | 5.38 | −36 | −75 | −12 |
| | | L Inferior temporal gyrus | 5.34 | −39 | −45 | −15 |
| | | L Lingual gyrus | 5.32 | −36 | −90 | −15 |
| 5 | 205 | L Inferior parietal lobule | 4.60 | −42 | −48 | 63 |
| | | L Inferior parietal lobule | 4.10 | −36 | −45 | 45 |
| 6 | 69 | **L Cerebellum, VIIB*** | 4.03 | −36 | −63 | −51 |
| | | **L Cerebellum, VIIIA** | 2.84 | −21 | −69 | −48 |
| 7 | 25 | **L Cerebellum, VIIAt*** | 3.34 | −9 | −75 | −30 |
| 8 | 34 | **L Cerebellum, Crus I*** | 3.13 | −45 | −45 | −33 |
| | | **L Cerebellum, VI** | 2.89 | −33 | −45 | −36 |
| | | **L Cerebellum, Crus I** | 2.72 | −45 | −54 | −33 |

Key: $R$ = Right; $L$ = Left. MNI coordinates = x, y, z coordinates of cluster peaks. Coordinates meeting voxel-level threshold of $p < 0.007$ and cluster-level threshold of $p < 0.05$ (corrected) are reported with the exception of * coordinates, which did not meet the cluster-level threshold.

Table 5
Viewing arousing vs. neutral images

| Cluster | Cluster size | Location | T-value | MNI coordinates | | |
|---|---|---|---|---|---|---|
| 1 | 1280 | R Middle temporal gyrus | 7.63 | 57 | −60 | 0 |
| | | R Middle temporal gyrus | 7.38 | 54 | −63 | 9 |
| | | R Middle temporal gyrus | 6.97 | 45 | −69 | 9 |
| 2 | 694 | L Inferior occipital gyrus | 7.06 | −48 | −75 | −6 |
| | | L Middle occipital gyrus | 6.73 | −48 | −75 | 3 |
| | | L Middle occipital gyrus | 6.15 | −42 | −75 | 12 |
| 3 | 36 | **L Cerebellum, VI*** | 3.97 | −42 | −45 | −24 |
| | | **L Cerebellum, VI** | 2.71 | −36 | −39 | −33 |
| 4 | 14 | **R Cerebellum, VI*** | 3.44 | 21 | −63 | −15 |

Key: $R$ = Right; $L$ = Left. MNI coordinates = x, y, z coordinates of cluster peaks. Coordinates meeting threshold of $p < 0.005$ and cluster-level threshold of $p < 0.05$ (corrected) are reported with the exception of * coordinates, which did not meet the cluster-level threshold.

Outside of the cerebellum, significant activation was found in large, bilateral frontal lobe clusters, and bilateral parietal clusters involving the precuneus on the right and the inferior parietal lobule and supramarginal gyrus on the left.

### *3.3.3. Verb generation*

Verb generation vs. Read only resulted in right cerebellar activation in lobules VI, VII and VIIIA; at certain points the lobule VI/Crus I cluster was separate from the Crus II/VIIB cluster (see Table 3).

Cerebral hemispheric activation was present in two large left-lateralized clusters: one frontal cluster extending from the medial to inferior frontal gyrus and including Broca's area, and one temporal-parietal cluster extending from the middle temporal gyrus to the superior parietal lobule, including the supramarginal gyrus. Smaller right-lateralized frontal and temporal-parietal activations were also observed.

### *3.3.4. Mental rotation*

The contrast of identifying the orientation of rotated vs. upright letters produced left cerebellar activation, with the largest cluster in lobule Crus II/VIIB extending into VIIIA, and the second largest cluster involving lobules VI and Crus I (Table 4). A medial cluster in lobule VII was evident between $x = -4$ and $x = -15$.

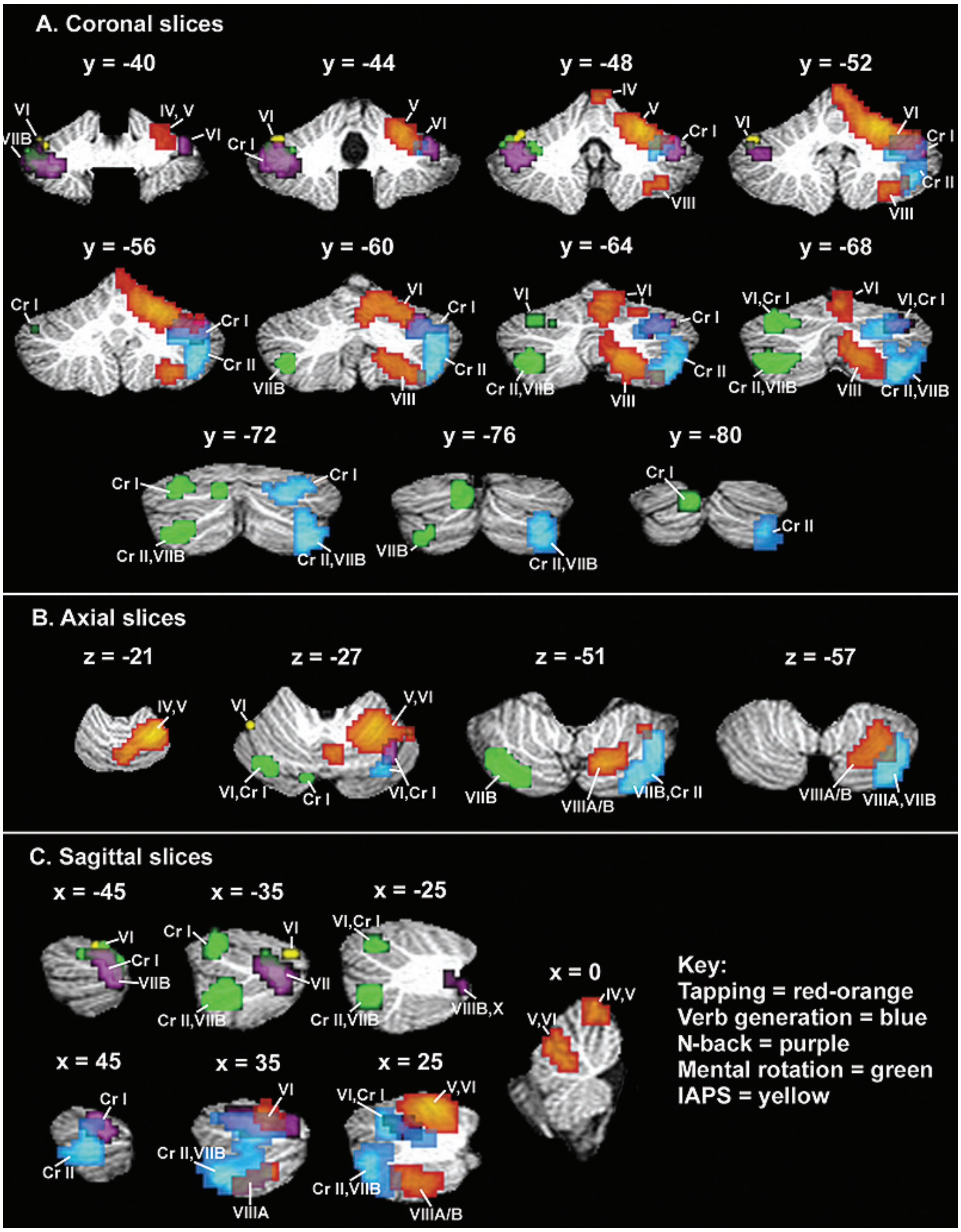

Fig. 1. (A) Coronal, (B) axial, and (C) sagittal slices through the cerebellum showing activation patterns for all tasks. Color-coding of activations produced by different tasks is in the key at bottom right. Left cerebellum is shown on the left in the coronal and axial slices; a negative x-value indicates left in the sagittal slices. Lobules are labeled according to nomenclature in [76]; Cr I, Crus I; Cr II, Crus II.

In the cerebral hemispheres there was a large right parietal-occipital cluster extending from the superior parietal lobule into occipital regions. Frontal activation was seen in the right superior and middle frontal gyri. Left-lateralized activation was evident in occipital-temporal, frontal, and inferior parietal areas.

### 3.3.5. *IAPS pictures*

Viewing emotionally arousing vs. neutral images activated small regions in cerebellar lobule VI bilaterally (Table 5).

In the cerebral hemispheres, a large right-lateralized cluster was present in the middle temporal gyrus. On the left, a smaller cluster involved inferior and middle occipital gyri.

### 3.4. Overlap between task activation patterns

There was a small amount of overlap in lobule VI between the tapping, verb generation and N-back tasks (e.g., see gray area in Fig. 1A, $y = -52$); motor-related activation extended into lobule VI from the anterior lobe, whereas activation during the cognitive tasks extended into lobule VI from lobule VII. There was also overlap between the motor and language measures in lobule VIIIA (see gray area in Fig. 1C, $x = 35$). In addition to these overlapping areas, the tapping- and cognitive-related activation patterns occupied distinct and separate locations in more posterior regions. Verbal working memory (N-back task) and verb generation showed overlapping activation in right lobule VI/Crus I, although the language-related activation also extended more inferiorly into lobule VIIB. The N-back cerebellar activation in Crus I in the left hemisphere overlapped with the activation related to mental rotation.

### 3.5. Reproducibility of activation patterns

Given the propensity for type I errors in the analysis of data from a single subject, we assessed how reproducible the activation patterns were within each domain by determining whether the activation level in a given cluster reached statistically significant levels during individual runs of the tasks (for tasks with two runs) or groups of blocks within a run (for tasks with one run). We extracted the data from each block from the clusters identified in the above analyses. The MarsBar Region of Interest (ROI) toolbox for SPM ([9]; http://marsbar.sourceforge.net/) was used to create ROIs of the cerebellar clusters for each task. For each cerebellar ROI per task, the T-statistics (and corresponding corrected $p$-values) were extracted from each block (with a "block" comprising a cycle of the task condition and the corresponding comparison condition).

For the tapping task, within the two cerebellar ROIs the T-values for each block were statistically significant (corrected $p$-values ranging from 0.000005 to 0.002), a robust outcome given the limited power inherent in sampling from individual blocks. During the N-back task, the 2-back vs. X-task contrast was significant for both ROIs in both runs (Crus I, run 1 $T = 2.87$, $p = 0.003$; run 2 $T = 5.26$, $p < 0.000001$; lobule VIIB, run 1 $T = 2.25$, $p = 0.013$; run 2 $T = 5.96$, $p < 0.000001$). The large right cerebellar cluster found during the verb generation task also showed significant activation during both runs (run 1 $T = 2.71$, $p = 0.0002$; run 2 $T = 5.86$, $p < 0.000001$). During the mental rotation task, all ROIs showed statistically significant T-values in run 1, but in run 2 only the Crus I ROI was statistically significant (VIIB: run 1 $T = 2.37$, $p < 0.000001$; run 2 $T = 0.16$, $p = 0.52$; VIIAt: run 1 $T = 1.62$, $p = 0.0002$, run 2 $T = 0.42$, $p = 0.24$; Crus I: run 1 $T = 1.51$, $p = 0.0009$, run 2 $T = 1.04$, $p = 0.02$). In the IAPS task, the single run was divided into odd and even blocks. The T-values were significant for both sets of blocks for the right ROI (odd blocks $T = 2.45$, $p = 0.008$, even blocks $T = 2.11$, $p = 0.02$), and were significant (odd blocks $T = 3.28$, $p = 0.0007$) and approached significance (even blocks $T = 1.37$, $p = 0.09$) for the left ROI.

## 4. Discussion

In this multiple-domain fMRI study we show that cerebellar functional topography is evident at the single-subject level. Tapping with the right index finger activated contralateral motor-related cerebral cortices along with ipsilateral cerebellar lobules IV-V extending into lobule VI and the second sensorimotor representation in lobule VIII. Activation during the working memory task was evident bilaterally both in the frontal and parietal lobes and in cerebellar lobules VII and VIIIA, as well as in right lobule VI. Verb generation activated left-lateralized frontal and parietal cortices and right cerebellar lobules VI, VII and VIIIA. Mental rotation engaged frontal and parietal-occipital regions (right more than left) and left cerebellar lobules VI, VII and VIIIA. Cerebellar activation was weakest in the IAPS task, with small bilateral clusters in lobule VI.

None of the non-motor tasks activated the anterior lobe of the cerebellum. Lobule VIIIA was involved in the majority of the tasks, but whereas motor activation spread from more medial lobule VIIIB to VIIIA, activation by the cognitive tasks extended into lobule VIIIA from the more laterally situated lobule VIIB. Similarly, the patterns of activation in lobule VI differed according to the motor or cognitive nature of the task. Lobule VI activation by motor tasks was part of a continuous cluster extending from lobules IV and V. In contrast, cognitive tasks produced clusters of activation confined to lobule VI, or involved lobule VI as part of a larger cluster that included lobule VII. Overlap was seen primarily between the regions involved in working memory and language (in the right cerebellum), and working memory and spatial processing (in the left cerebellum).

### 4.1. Correspondence with other neuroimaging studies

In this single subject, activation patterns corresponded well with the results of our meta-analysis of cerebellar activation patterns reported in previous neuroimaging studies [88]. In particular, cerebellar activation sites related to finger tapping overlapped with the regions identified for motor and somatosensory processing (e.g., the peak in lobule VIIIA at +15 −66 −48 in this subject corresponds to the peak for somatosensory processing in the meta-analysis at +18 −62 −46). The finger-tapping results of the current study are also compatible with previous neuroimaging findings investigating the topographic organization of sensorimotor function in the cerebellum [14,29,68].

Language-related activation during phonological, semantic and word generation paradigms has been observed in the right posterolateral cerebellum, with more involvement of the anterior lobe when articulation is a factor (see [23]; and clinical studies [1]). The activation peak found in right lobules VI/Crus I during verb generation in our subject is consistent with the lobule VI/Crus I peaks found when the activation patterns of various types of language tasks were combined in the meta-analysis [88]. Notably, along with right cerebellar regions, the left cerebral hemisphere language network regions were engaged by the verb generation task. These findings are compatible with studies of cerebro-cerebellar language circuits, which indicate that the contralateral posterior cerebellar hemisphere is engaged in subjects with left- or right-lateralized language dominance (e.g. [38,41,100]).

Two early studies of mental rotation reported strong cerebellar activation [8,64]. In this study, the cerebral activation during mental rotation was bilateral, though stronger in the right hemisphere, the cerebellar activation was left-lateralized and localized to lobules VI and VII, extending into lobule VIIIA. Similarly, other studies investigating mental transformations have found that cerebral activation is more bilateral than in language tasks, though cerebellar activation tends to be stronger in the left cerebellar hemisphere (e.g. [94,95,99]). The left cerebellar regions active in our study also correspond well to the meta-analysis findings, particularly the peaks in left lobules VIIAt and VI.

Early imaging studies highlighted the role of the cerebellum in working memory (e.g. [18,24]). In these and subsequent analyses, working memory paradigms activated bilateral regions of the cerebellar posterior lobe ([36,45,92,93]; see [88]). The results of our current study and previous meta-analysis show that regions involved in verbal working memory tasks overlap with those involved in language.

Viewing emotional vs. neutral images from the IAPS scale [47] is known to activate cerebellar regions in the posterior lobe, including lobules VI and VII [6,27,34,39,46,48,61–63,90]. In our case, the IAPS task produced activation in lobule VI bilaterally, stronger on the left, but the posterior vermis activation that we predicted was not evident. The IAPS task also produced the weakest cerebellar activation in this study. There are several potential explanations for these findings. We performed only one run of this task, which may have resulted in insufficient power. Negative stimuli activate the cerebellum (and occipito-temporal regions) more significantly than positive stimuli [46,62,90], and the combination of positive and negative arousing stimuli used in this study also may have limited the cerebellar activation. It is worth noting that the task also did not activate traditional limbic regions [60], such as the cingulate gyrus, amygdala, hippocampus, or hypothalamus. These data do, however, provide further evidence for lobule VI activation by emotionally salient stimuli. This is consistent with previous studies showing bilateral lobule VI activation in tests of emotional perception [6,46] and of empathy for another's pain [84].

### 4.2. Implications

The likely basis of the functional topography of the cerebellum identified in this and earlier fMRI studies is the existence of multiple cerebro-cerebellar loops, identified in anatomical and physiological studies in experimental animals, that appear to target different pontine and cerebellar regions depending on their cerebral connections [3,10–12,37,43,71,73,77–80]. Together, these studies provide evidence that, whereas the motor cortex is linked with cerebellar lobules IV and V with a small involvement of lobule VI, prefrontal and posterior parietal cortices are reciprocally interconnected with cerebellar lobule VII and its hemispheric extensions Crus I and Crus II.

Our findings support the relationship between activation in the sensorimotor cortices and lobules IV-VI, and frontal/parietal activation in concert with Crus I/II activation. The degree to which the Crus I/II connections are specific for these different cerebral association areas has not been established. In our single-subject study, right Crus I was active during verb generation; right Crus I and left Crus II were active during the N-back task; and left Crus I was highlighted during mental rotation. For these tasks, however, cortical activations were present in both frontal and parietal regions. This makes it difficult to discern whether these imaging data indicate the existence of separate prefrontal- vs. parietal-cerebellar anatomical loops.

A central unresolved question regarding topographic organization of function in the cerebellum is the relationship between the anatomical topography of connections with cerebral association cortices and the functional topography within the cerebellum of the cognitive domains subserved by these cerebral association areas. We have previously proposed rules governing the relationship between the cerebellum and cognitive processing [72], including the convergence of inputs from multiple associative cerebral regions to common areas within the cerebellum, thus facilitating cerebellar regulation of supramodal functions. The dispersed nature of the cerebral cortical input to the pons from associative and paralimbic cortices as well as from the sensorimotor cortices suggests that cerebellar cortical terminations arising from prefrontal areas may be found in the cerebellum in areas adjacent to inputs from parietal or superior temporal polymodal areas, or to those from paralimbic cortices in the cingulate and posterior parahippocampal gyri. But connections of specific cerebral areas with specific cerebellar regions are still not adequately established, and therefore this remains an open question. The direct clinical relevance of this is that cerebellar clinical syndromes manifest traces of different "cerebral" behavioral syndromes, as exemplified by the CCAS resulting from posterior lobe lesions, which includes impairments in executive, visual-spatial, linguistic and affective domains.

### *4.3. Limitations and caveats*

Because our study was designed to measure task-related activation in five different measures in one functional imaging session, we employed a block design to provide more power. This restricted our capacity to interpret which specific aspects of these tasks require cerebellar circuitry. A related caveat is that, due to our requirement that a single subject perform all tasks during one session, a limited number of measurements were obtained for each task. For the majority of tasks, this was not problematic, but it is possible that the one run of the IAPS task provided insufficient power to elucidate all but the greatest cerebellar activation peaks. It is also important to note that the risk of type I errors is increased in this single-subject study. In terms of task selection, our goal was to explore the functional patterns found within the cerebellum in a single subject, and thus we were limited to tasks that reliably activate the cerebellum in neuroimaging studies. Further, while we employed tasks that engage different cortical regions, and can make deductions about cerebro-cerebellar networks, we have not directly measured or isolated these networks. Finally, although these results are consistent with previous findings, as with all single-subject studies there is the question of reproducibility and generalization, and whether the same activation patterns will be seen both within the same subject at a different point in time and in other subjects.

### *4.4. Conclusions and future directions*

To our knowledge, this is the first fMRI study in which multiple sensorimotor and cognitive domains were examined within a single individual. Our results provide the necessary evidence to establish the proof of principle of topographic organization of motor, cognitive and emotional processing in the cerebellum at the level of a single subject. The correspondence between our single-subject results and the published functional imaging literature indicates that – despite differences in task paradigms, image acquisition, and analysis – cerebellar activation patterns are fairly consistent across subjects and studies, and can be identified at a single-subject level. Further, overlap in cerebellar lobules between sensorimotor and cognitive tasks rarely occurred. The presence of some degree of overlap between the activation patterns for the different cognitive tasks may reflect shared cerebellar substrates for cognitive domains that recruit similar cerebro-cerebellar circuits. Future research is needed to establish the intra-subject consistency across time, and inter-subject variability of these findings; to determine which cerebro-cerebellar circuits might drive these activation patterns; and to investigate the cerebellar contribution to these different functional domains. This demonstration of functional topography in the human cerebellum has relevance for understanding motor and non-motor manifestations of cerebellar lesions, and for predicting and ultimately treating motor, cognitive and affective disturbances resulting from disorders of the cerebellum.

## Acknowledgements

This study was supported in part by The National Center for Research Resources (P41RR14075); the Massachusetts General Hospital Fund for Medical Discovery (CJS); the National Institutes of Health (071535, EMV); and the Birmingham Foundation and MINDlink Foundation (JDS). The authors would like to thank Larry Seidman for the use of the N-back task, Peter Hansen for providing stimuli for the mental rotation task, Janet Sherman for help with the psychometric test battery, and Scott Slotnick for helpful discussion.
